# A vessel length-based method to compute coronary fractional flow reserve from optical coherence tomography images

**DOI:** 10.1186/s12938-017-0365-4

**Published:** 2017-06-26

**Authors:** Kyung Eun Lee, Seo Ho Lee, Eun-Seok Shin, Eun Bo Shim

**Affiliations:** 1 Department of Mechanical and Biomedical Engineering, Kangwon National University, Kangwondaehak-gil, Chuncheon-si, Kangwon-do 200-701 Republic of Korea; 2 Department of Cardiology, University of Ulsan College of Medicine, Ulsan, South Korea

**Keywords:** Fractional flow reserve, Optical coherence tomography, Computer simulation, Vessel length-based method

## Abstract

**Background:**

Hemodynamic simulation for quantifying fractional flow reserve (FFR) is often performed in a patient-specific geometry of coronary arteries reconstructed from the images from various imaging modalities. Because optical coherence tomography (OCT) images can provide more precise vascular lumen geometry, regardless of stenotic severity, hemodynamic simulation based on OCT images may be effective. The aim of this study is to perform OCT–FFR simulations by coupling a 3D CFD model from geometrically correct OCT images with a LPM based on vessel lengths extracted from CAG data with clinical validations for the present method.

**Methods:**

To simulate coronary hemodynamics, we developed a fast and accurate method that combined a computational fluid dynamics (CFD) model of an OCT-based region of interest (ROI) with a lumped parameter model (LPM) of the coronary microvasculature and veins. Here, the LPM was based on vessel lengths extracted from coronary X-ray angiography (CAG) images. Based on a vessel length-based approach, we describe a theoretical formulation for the total resistance of the LPM from a three-dimensional (3D) CFD model of the ROI.

**Results:**

To show the utility of this method, we present calculated examples of FFR from OCT images. To validate the OCT-based FFR calculation (OCT–FFR) clinically, we compared the computed OCT–FFR values for 17 vessels of 13 patients with clinically measured FFR (M-FFR) values.

**Conclusion:**

A novel formulation for the total resistance of LPM is introduced to accurately simulate a 3D CFD model of the ROI. The simulated FFR values compared well with clinically measured ones, showing the accuracy of the method. Moreover, the present method is fast in terms of computational time, enabling clinicians to provide solutions handled within the hospital.

## Background

Coronary artery imaging is valuable for the diagnosis of coronary artery disease. Invasive and non-invasive imaging techniques, such as coronary X-ray angiography (CAG), computed tomography (CT), magnetic resonance imaging (MRI), optical coherence tomography (OCT), and intravascular ultrasound (IVUS), enable the visualization of the arterial lumen, plaque, and vascular wall structures [1].

Anatomical data extracted with such imaging techniques have been used for computer simulations to assess the physiological or functional severity of coronary stenotic lesions. As an example, Kim et al. [2] proposed a simulation method to compute the fractional flow reserve (FFR) of the coronary arteries using CT images from patients [2]. In their study, a computational fluid dynamics (CFD) technique was used to simulate the blood flow in the three-dimensional (3D) geometry of the coronary arteries. To specify the outlet boundary conditions for the CFD model, they used a lumped parameter model (LPM) to reflect the influence of the coronary microvasculature and venous system. It is important to estimate the resistance values in the LPM. For this purpose, they measured the muscle mass quantity, fed by a specific coronary artery, from which the resting blood flow in the artery was estimated, based on a scaling law [3–5]. Then, the resistance value of the artery can be computed from the resting flow. For this reason, the 3D muscle mass of the left ventricle must be determined to use this method, limiting its application to CT imaging.

Recently, high-resolution OCT imaging has been used to calculate coronary hemodynamics in image-based simulation models [6–10]. In these studies, the local region of interest (ROI) of the coronary artery, scanned using OCT, was merged into a rough 3D coronary arterial model reconstructed from biplane CAG images [10–14]. Then, CFD simulation was used with the combined model to calculate the FFR, wall shear rate, and wall shear stress [6–10]. However, these studies have a limitation. Unlike CT image-based FFR simulations [2–5], the muscle mass to specify the resistance values of the LPM could not be determined from the OCT and CAG images, and thus the studies could not construct the LPM. Thus, the flow behavior in these OCT-based models was simply simulated using a fully developed flow condition in the outlet, without considering the effects of microvascular and venous hemodynamics [6–10]. The purpose of this study is to present an efficient and accurate method for performance of OCT–FFR simulations by coupling a 3D CFD model from geometrically correct OCT images with a LPM based on vessel lengths extracted from CAG data.

**Fig. 1:**
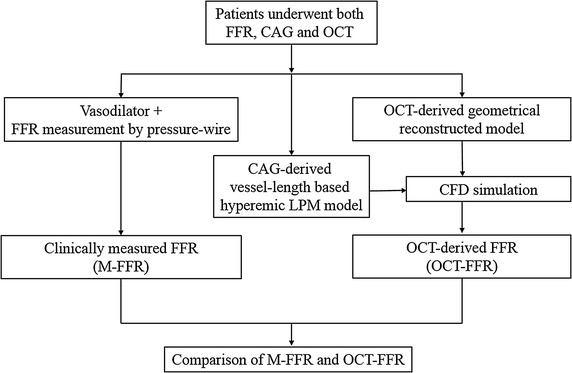
Flowchart of study design. FFR, fractional flow reserve; M-FFR, clinically measured FFR; OCT–FFR, fractional flow reserve calculated from OCT; CAG, coronary X-ray angiography; LPM, lumped parameter model; and CFD, computational fluid dynamics

To simulate the coronary FFR distribution, we describe here a new method that couples a geometrically correct 3D CFD model, reconstructed from OCT images, with a LPM to reflect coronary microvascular and venous hemodynamics. The resistance of the LPM was estimated using a simplified formulation based on the vessel lengths of the coronary arterial tree in CAG images. To demonstrate the validity of the method, we performed an OCT image-based FFR simulation (OCT-FFR simulation) for 17 vessels from 13 patients and compared the results with clinically measured FFR data (M-FFR), according to the protocol in Fig. 1.

## Methods

### Clinical measurement and geometric model

**Fig. 2:**
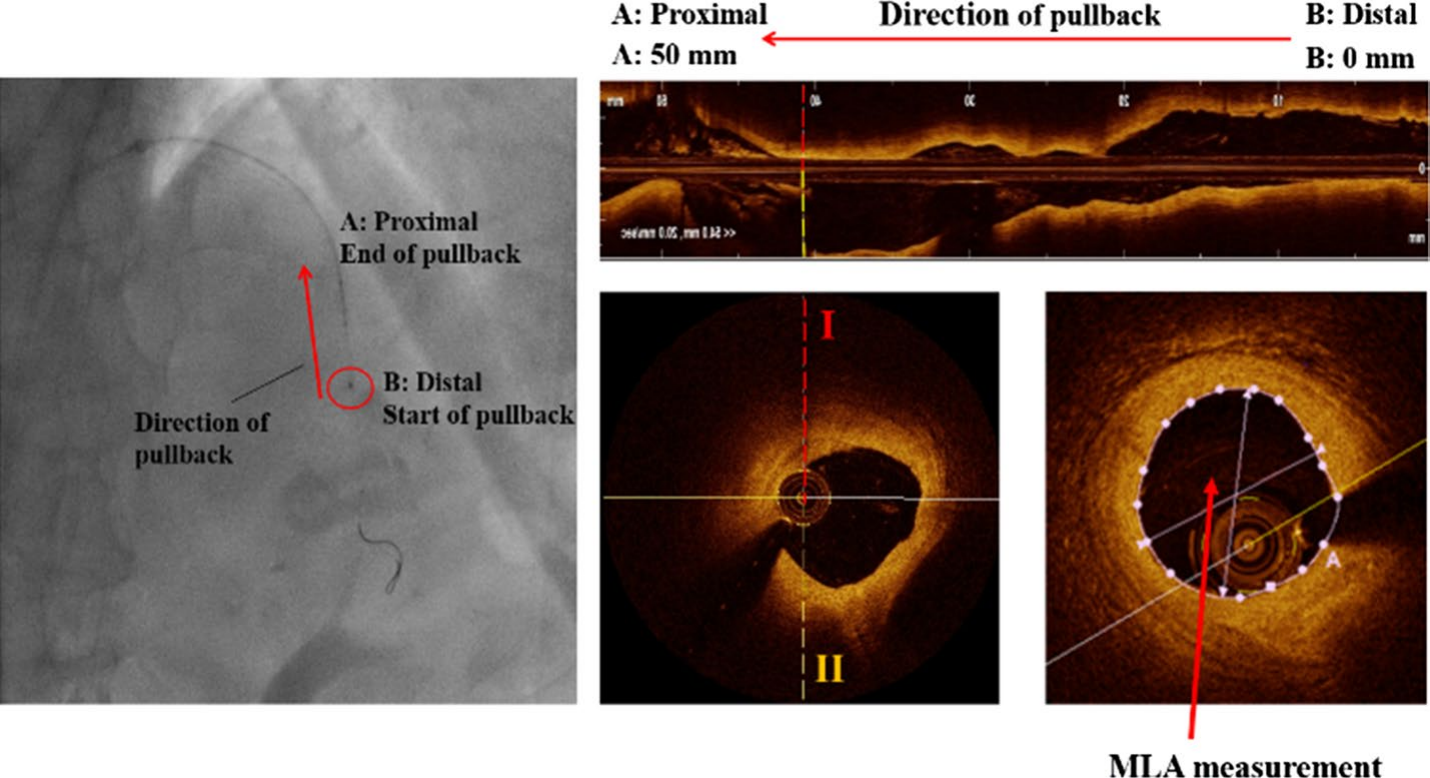
Regions of interest (ROI) in target vessels were scanned by OCT during pullback from *B* to *A*

We simulated the coronary hemodynamics of 17 vessels from 13 patients who underwent CAG, OCT, and FFR measurement at Ulsan University Hospital (Ulsan, Republic of Korea) between August 2015 and June 2015. The CAG, OCT, and FFR examinations followed the current guidelines [15–17]. The target ROI of a coronary vessel was scanned by OCT after administration of 200 μg intracoronary nitroglycerine. Images were acquired using a commercially available frequency-domain OCT system and optical catheter (C7-XR imaging system and Dragon Fly catheter; Lightlab Imaging/St. Jude Medical, Westford, MA, USA; Fig. 2). FFR was measured in a hyperemic state after the administration of an adenosine stimulus [16]. Figure 2 shows the distal start and proximal end points of the OCT pullback to scan the ROI of the coronary artery.

For construction of the 3D CFD model, OCT image slices were segmented in a semi-automated method using open-source software (ITK-snap; ver. 2.4). Then, we integrated the segmented slices for the 3D volume construction of the target ROI. A 3D tetrahedral volume mesh was generated using Delaunay’s triangulation algorithm and a triangular prismatic mesh layer was inserted into the boundary of the target ROI.

### Computational method

In the present scheme, the LPM, reflecting coronary microvascular and venous hemodynamics, was connected to the outlet boundary of the 3D CFD model of the target ROI. Estimation of the LPM parameters was based on the vessel length-based method proposed in our previous paper [18], but we applied the method to the model with the short 3D vessel geometry of the target ROI.

**Fig. 3:**
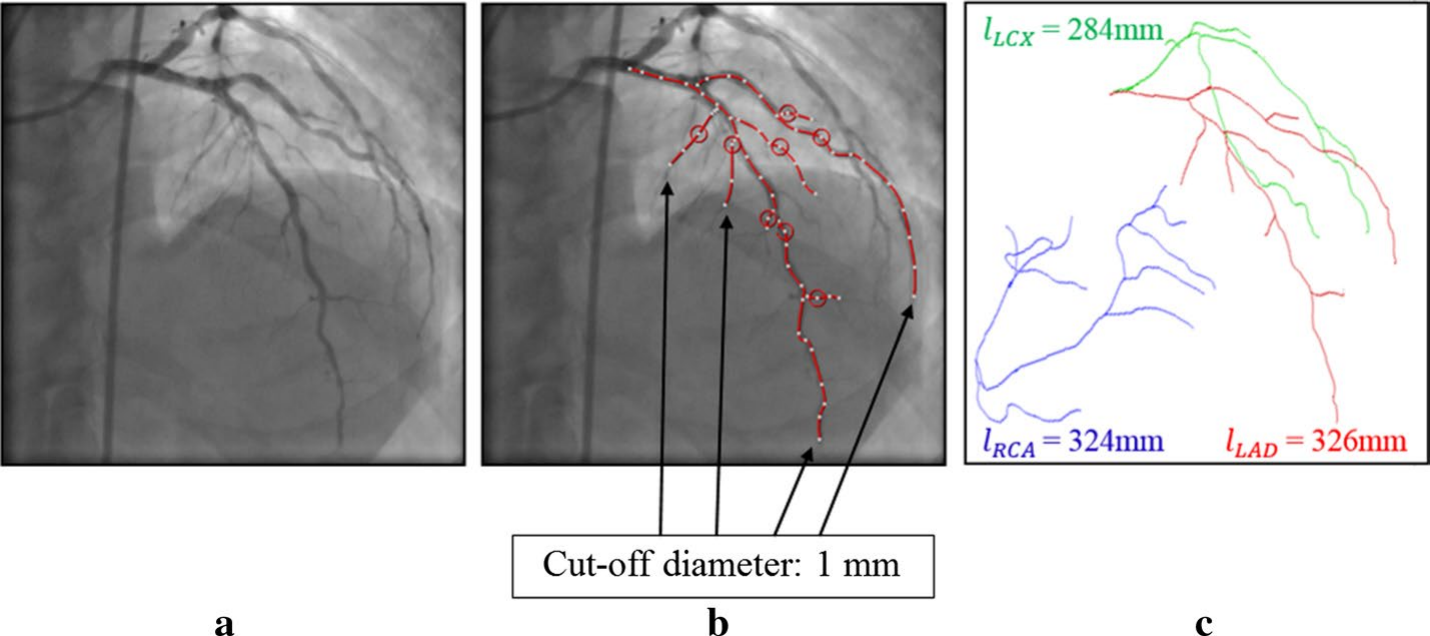
Length of the vessels ($l_{LAD} , \;l_{LCX} ,\; l_{RCA} )$ was measured by CAG. The lengths of LAD, LCX, and RCA are indicated in *red*, *green*, and *blue*, respectively. The cut-off diameter was 1 mm. LAD, left anterior descending artery; LCX, left circumflex artery; RCA, right coronary artery. **a** Arterial trees on coronary X-ray angiography (CAG). **b** The cut-off diameter of vessel-length measurement for OCT-FFR simulation. **c** The lengths of LAD, LCX, and RCA

Similar to our previous report [18], we first calculated the resistance values of the right coronary artery (RCA), left anterior descending artery (LAD), and left circumflex artery (LCX) from the vessel lengths of the vascular tree, the centerlines of which were extracted from CAG images. Figure 3 shows an example of the estimated vessel lengths extracted from a CAG image; using the CAG image, we first identified the vessel segment lengths where the diameter exceeded 1 mm (Fig. 3a, b). To calculate the vessel length of the LAD, we summed the lengths of the LAD sub-vessels, the diameters of which were greater than a specific cut-off value (here, 1 mm; Fig. 3c). In our experience, 1 mm is a suitable value for the diameter cut-off; however, this can be changed depending on the quality of the CAG images. After identification of the vessel lengths of LAD, LCX, and RCA ($$l_{LAD}$$, $$l_{LCX}$$, and $$l_{RCA} )$$, the flow division ratio to the branches can be specified as follows [18]:

1 $$Q_{LAD}{:}Q_{LCX}{:}Q_{RCA} = l_{LAD}{:}l_{LCX}{:}1/\left\{ {\alpha /(l_{RCA} )_{RV} + 1/(l_{RCA} )_{LV} } \right\},$$

where $Q$ is the flow rate, $l_{LAD}$ and $l_{LCX}$ are the vessel lengths of the LAD and LCX, $\left( {l_{RCA} } \right)_{RV}$ and $\left( {l_{RCA} } \right)_{LV}$ are the vessel lengths of the RCA feeding the right ventricle (RV) and left ventricle (LV), respectively, and $\alpha$ is the volume ratio of LV to RV. As described in our previous paper [18], Eq. (1) has two noteworthy points. First, a longer vessel feeds more muscle mass, inducing more blood flow and thus less flow resistance. Second, RCA vessels feed not only RV muscle but also LV muscle. However, the RV muscle layer is much thinner than that of the LV, so the lengthwise contribution of RV feeding vessels to RCA blood flow induction is less than that of the LV feeding ones. This was the reason we introduced the volume ratio of LV to RV, $\alpha$. We assumed the value of $\alpha$ to be 3.46, a typical value observed in clinical trials [19]. If we assume the total coronary flow is 4% of cardiac output and the pressure difference of the whole coronary circulation is $\Delta P$, then the resting flow rates to LAD, LCX, and RCA can be obtained and thus the resistance values exerted in LAD, LCX, and RCA (*R*_*LAD*_, *R*_*LCX*_, and *R*_*RCA*_, respectively) can be calculated sequentially. Here, $\Delta P$ can be obtained if the aortic pressure and right atrium pressure are specified. In this study, the aortic pressure was estimated from the brachial pressure of a patient and the right atrium pressure was set at 5 mmHg, as in a previous report [20].

**Fig. 4:**
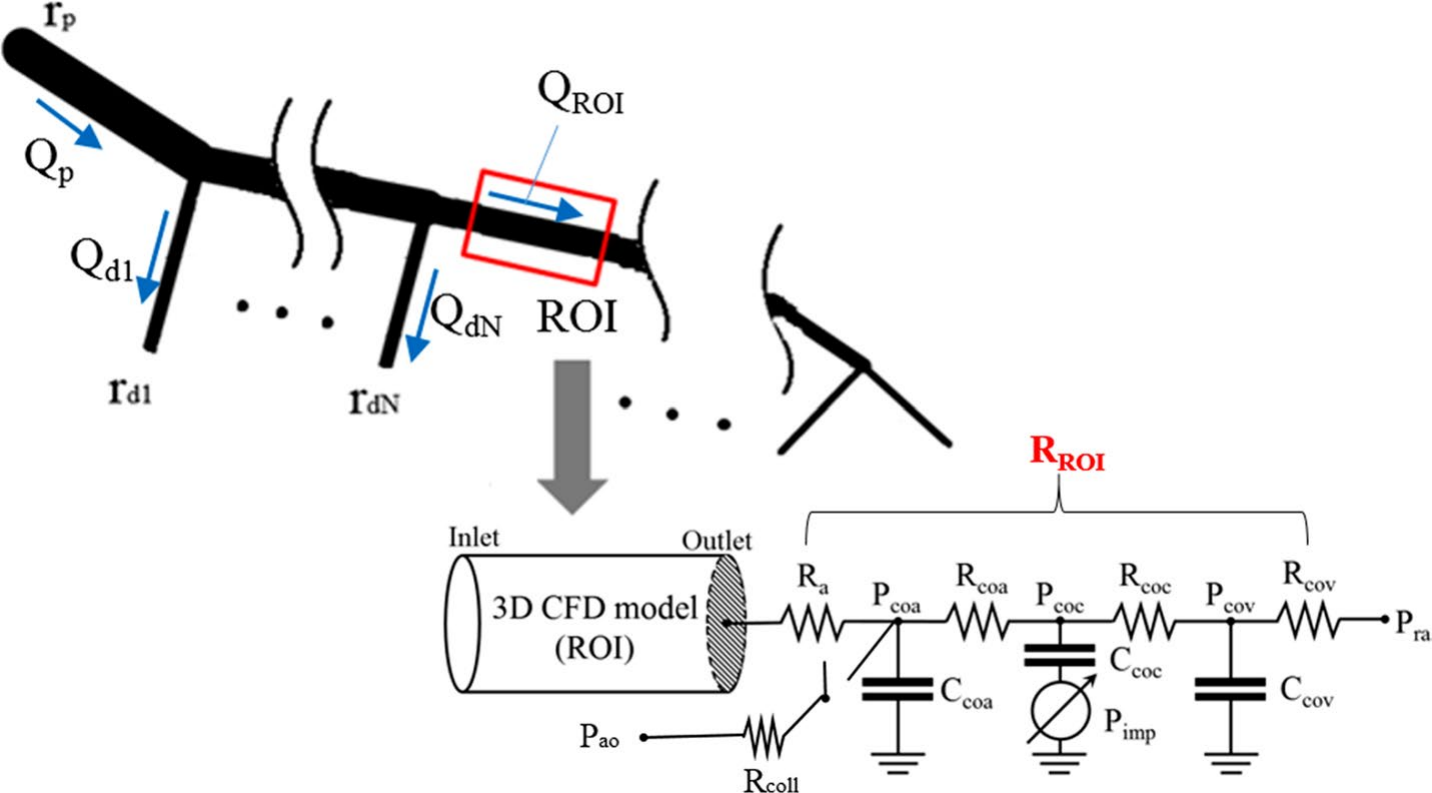
Schematic diagram of the present method. The OCT-derived three-dimensional (3D) ROI model was coupled with a length-based LPM from CAG. Here, *Q*, flow rate; *P*, pressure; *R*, resistance; *C*, capacitance; *r*, radius of the vessel segment. *Subscripts*, ao, aorta; a, large artery; coa, coronary arterioles; coc, coronary capillaries; cov, coronary veins; imp, intra-myocardium; coll, collateral; ra, right atrium

For the given values of the resistances *R*_*LAD*_, *R*_*LCX*_, and *R*_*RCA*_, we can determine the resistance value of the microvasculature and veins after the outlet of the 3D ROI reconstructed from OCT images. Figure 4 shows a schematic diagram of a coronary vascular tree and ROI. If we assume that a parent vessel is divided into *N* sub-tree vessels and the vessel of the ROI, the following equation can be derived from the flow conservation principle:

2 $$Q_{P} = Q_{d1} + \cdots + Q_{dN} + Q_{ROI}$$

Here, $Q_{P}$ is flow in the parent vessel, meaning the total blood flow through one of the main coronary branches, such as RCA, LAD, and LCX. $Q_{di}$ is the flow of the *ith* vessel branch among the daughter vessels; the subscript “*i*” ranges from *1* to *N*, the total number of daughter branches before the ROI. $Q_{ROI}$ is the flow through the 3D ROI. A scaling relationship between lumen radius and blood flow rate is valid in each branch [21, 22].

3 $$Q = m \cdot r^{k} ,$$

where *r*, *m*, and *k* are the radius of a vessel segment, a proportionality constant, and the exponent, depending on flow conditions, respectively. The generally used value of *k* is ~2–3 [4]. In this study, we used *k* = 2 to reflect the dependency of flow rate on vascular cross-sectional area [20, 23]. Inserting the flow–radius relationship of Eq. (3) into Eq. (2), we derive the following equations:

4 $$Q_{ROI} = Q_{p} - m \sum \limits_{i = 1}^{N} r_{di}^{k} = {\text{${\Delta P}$} /\text{${R_{ROI} }$}},$$

5 $$R_{ROI} = \Delta P \left/\left( {Q_{p} - m\mathop \sum \nolimits_{i = 1}^{N} r_{di}^{k} } \right)\right. .$$

Here, $\Delta P$ is the pressure difference between the inlet of the ROI and the end of coronary circulation, right atrium. We also used the following equation:

6 $$Q_{p} = m r_{p}^{k} \to m = {Q_{p} } /{r_{p}^{k} }.$$

If we insert this expression for *m* into Eq. (5) and consider the relationship between flow and pressure, we derive the final form of $R_{ROI}$:

7 $$\begin{aligned} R_{ROI} &= \Delta P \times r_{p}^{k} \left/ Q_{p} \left( {r_{p}^{k} - \mathop \sum \nolimits_{i = 1}^{N} r_{di}^{k} } \right)\right. = \left( {r_{p}^{k} \times R_{p} } \right)\left/\left( {r_{p}^{k} - \mathop \sum \nolimits_{i = 1}^{N} r_{di}^{k} } \right)\right. \hfill \\ &= R_{p} /\left\{ {1 - \mathop \sum \nolimits_{i = 1}^{N} \left( {r_{di} /r_{p} } \right)^{k} } \right\} =\, R_{p} /AMP_{R} \quad (0 \le AMP_{R} \le 1) \hfill \\ \end{aligned}$$

In Eq. (7), *R*_*p*_ is one of the resistances, *R*_*LAD*_, *R*_*LCX*_, or *R*_*RCA*_. *AMP*_*R*_ indicates the amplification factor of the resistance and is determined from *r*_*p*_ and *r*_*di*_ (*i* = *1*,…,*N*) that are estimated from the analysis of CAG images. Then, we can calculate the resistance of the microvasculature and veins after the ROI (*R*_*ROI*_) vessel.

In the present study, the LPM model comprises a resistor, a capacitor, and the intra-myocardial pressure (Fig. 4) [23, 24]. *R* is the resistance (blood flow resistance), *P* is the pressure, and *C* is the capacitance (vessel compliance). The subscripts indicate the circulation component: ao, aorta; coa, coronary arteries; coc, coronary capillaries; cov, coronary veins; ra, right atrium; imp, intra-myocardium; and coll, collateral effects. To consider the influences of compression of the left ventricular muscle around the intramuscular vessels, we assumed $P_{imp} = \gamma \cdot P_{LV}$, where $P_{LV}$ is the left ventricle pressure, with ${{\upgamma }} = 0.75$ [23]. The capacitance values used in the LPM were those suggested in a previous paper [25], and the total resistance value of the ROI, *R*_*ROI*_, was distributed among coronary sub-compartments, such as the coronary arteries, coronary capillaries, and coronary veins, as suggested previously [23]. The collateral influence was included using the switching system in Fig. 4. The on–off switch remains open when coronary arterial pressure *P*_*coa*_ is beyond a specific level [26, 27]. However, when severe stenosis causes too large a pressure drop and thus *P*_*coa*_ decreases to the specific level, the switch is closed, inducing collateral flow. This collateral flow is regulated by the collateral resistance, *R*_*coll*_ (Fig. 4) [26].

To couple the LPM of the coronary circulation with the OCT-based CFD model, we used an iterative method in each time step, in which the computed flow rate in the outlet of the CFD model was transferred to the LPM. Then, we calculated the pressure and flow rates in the LPM by solving the ordinary differential equations. The computed value of the coronary arterial pressure in the LPM was used for the CFD model as the outlet boundary conditions of the CFD model. Namely, the established LPM model was then coupled with a 3D CFD coronary model by iteratively updating pressure and flow rate until interfacial boundary consistency. Then, computed FFR distributions can be obtained in the 3D ROI of the coronary arterial model. A more detailed description of the coupling method is found in our previous paper [23].

To simulate hyperemic flow condition in the model, we reduced the coronary resistance value to 25% of normal, based on an adenosine infusion experiment [28]. The time varying component of arterial resistance was not considered in the present method because it is minimized at hyperemic state.

### Flow conditions and governing equations

Blood was assumed to be a laminar, viscous, and Newtonian fluid. Turbulence influences when Re ≥ 3000 were neglected because blood flow in coronary arteries was laminar with Re ≤ 2000 [29–31]. The patient-specific viscosity was calculated using the Einstein model with a patient-specific hematocrit (HCT) (Table 1) [23].

**Table 1:** Patient clinical data

Parameter	Clinical data
Age (years)	57.5 (51.75–63.5)
Weight (kg)	70.5 (61.5–73.5)
Systolic blood pressure (mmHg)	140 (122–150)
Diastolic blood pressure (mmHg)	81 (66–91)
Heart rate (bpm)	67 (56–71)
Stroke volume (mL)	65 (56–74.6)
Hematocrit (%)	40.2 (38.1–46)

Number of patients, *n* = 13, number of ROI, *n* = 17. Data are provided as median values (interquartile range)

In the CFD model, the flow behavior of incompressible blood flow is governed by the Navier–Stokes equations, as in Eqs. (8) and (9):

8 $$\nabla \cdot \varvec{u}\text{ = 0}$$

9 $$\partial \varvec{u}/\partial t + \varvec{u} \cdot \nabla \varvec{u} = - \nabla p/\rho + {{\upmu }}/\rho \;\nabla^{2} \varvec{u}$$

Here, ***u***, *t*, $\rho$, ${{\upmu }}$ are the velocity vector, time, fluid density, and viscosity, respectively. These equations can be solved using a segregated finite-element method as described previously [23].

## Results

**Fig. 5:**
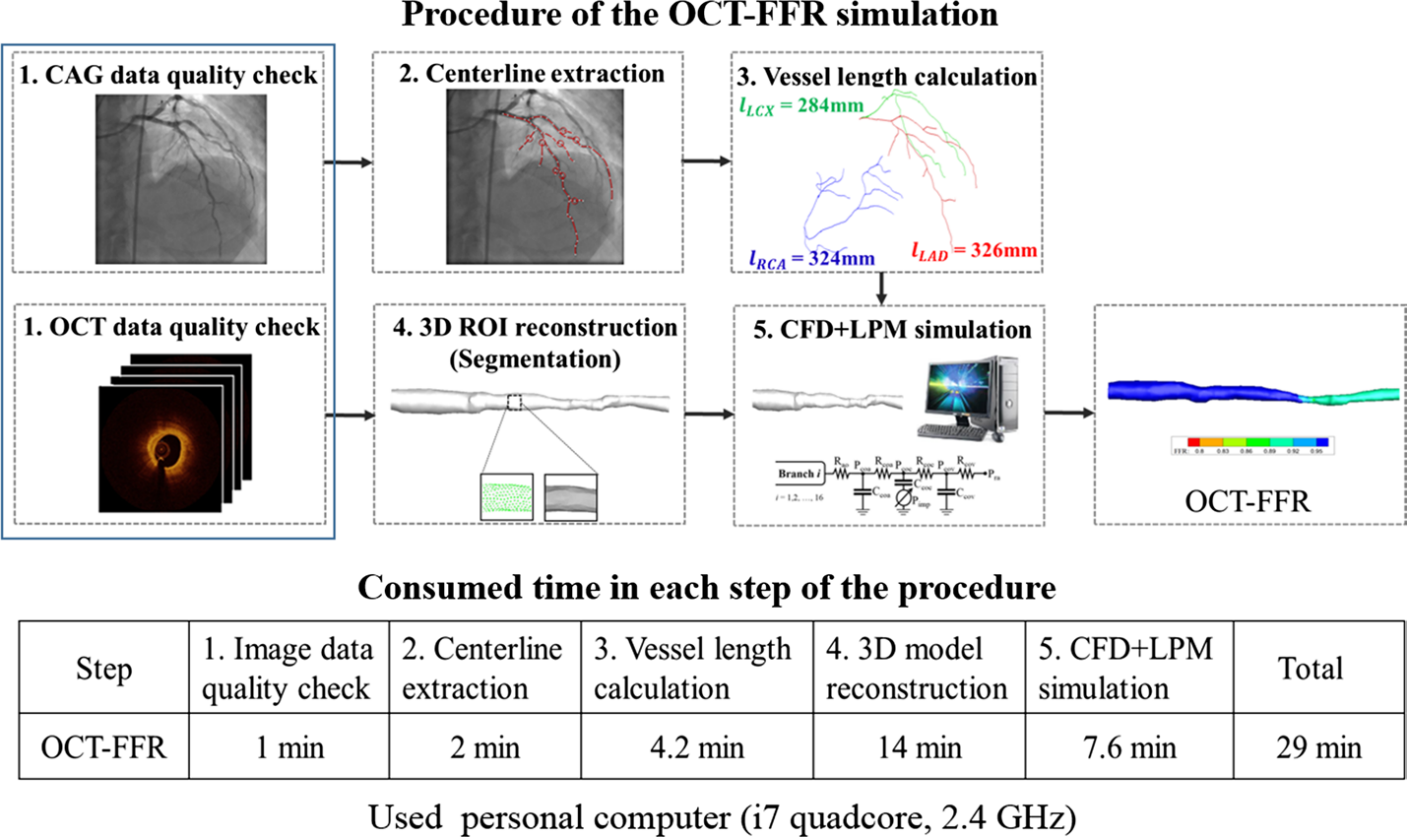
Computational procedure of the present OCT–FFR simulation and time consumed in each step

To validate the method described, we simulated 17 vessels of 13 patients who underwent CAG, invasive FFR, and OCT. The length-based flow distributions in the coronary arteries of the 13 patients are shown in Table 2. The mean flow distributions in the LAD, LCX, and RCA were 33, 30, and 37%, respectively, for the 17 vessels of the 13 patients. As shown in Fig. 5, the present OCT-FFR simulation consisted of five steps and the total computational time for a case was ~29 min on a typical personal computer, enabling clinicians to make on-site decisions for patients.

**Table 2:** Estimates of flow distributions in LAD, LCX, and RCA by a length-based method using X-ray angiography (CAG)

	Q_LAD_ (%)	Q_LCX_ (%)	Q_RCA_ (%)
Case 1	30	30	41
Case 2	35	30	35
Case 3	19	26	55
Case 4	34	31	35
Case 5	19	36	46
Case 6	35	31	34
Case 7	36	31	33
Case 8	25	36	39
Case 9	44	25	31
Case 10	40	23	36
Case 11	34	33	33
Case 12	34	37	38
Case 13	38	26	36
Mean	33	30	37

**Fig. 6:**
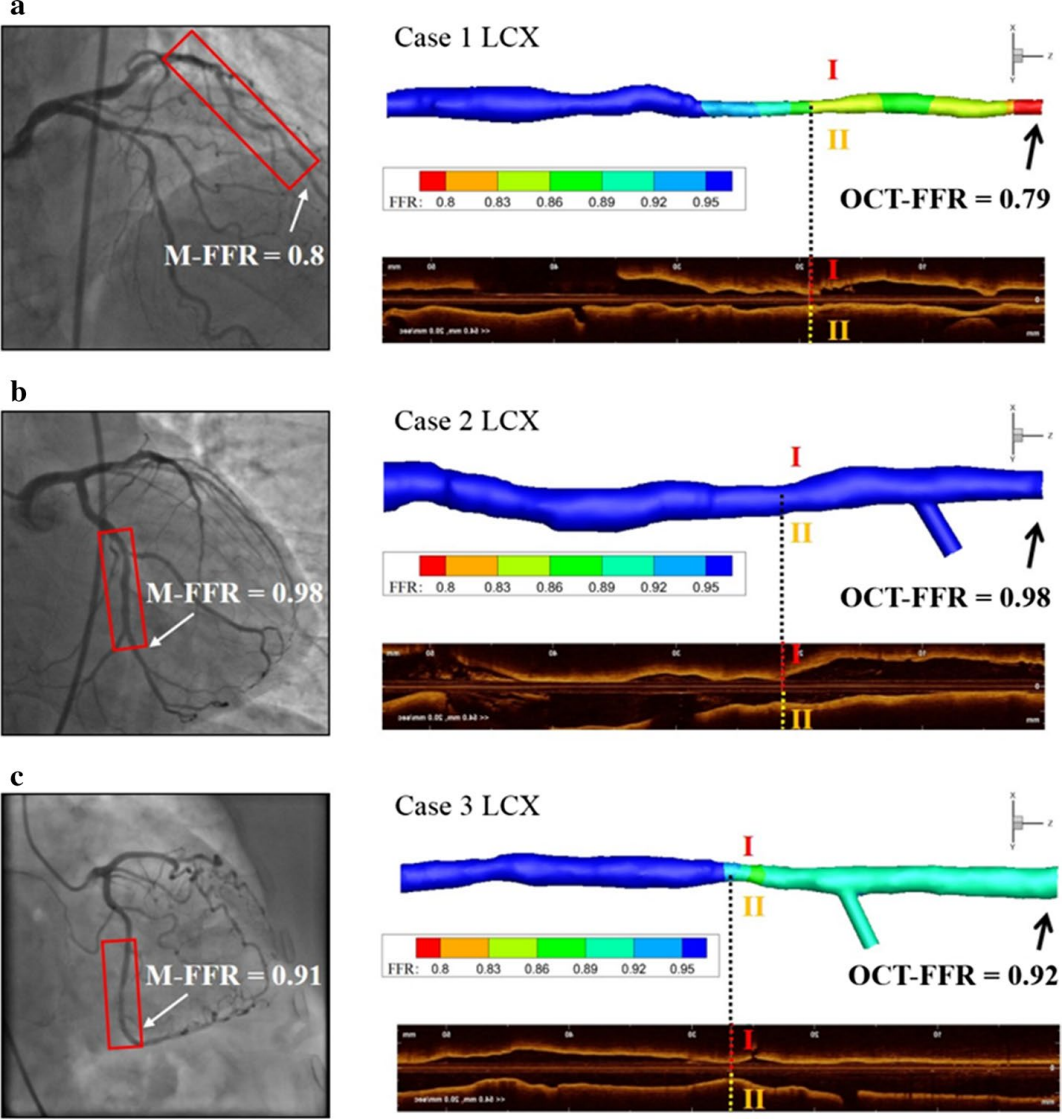
Examples showing comparisons between the measured FFR values (*left panel*), and the computed FFR ones, and OCT images from proximal to distal OCT images (*right panel*) for three patients with relatively severe stenosis (**a**), almost no stenosis (**b**), and relatively mild stenosis (**c**)

We compared the computed OCT–FFR values with M-FFR data measured with a pressure sensor during invasive pressure wire insertion for the 17 vessels of the 13 patients. Representative examples are shown for comparison; three cases of the LCX from three patients among the 17 vessels were chosen (Fig. 6). Figure 6 shows the OCT-scanned ROI region in the red box, M-FFR values with the measured locations using a white arrow on the angiographic images (left panel of Fig. 6), and the computed OCT–FFR contours (right panel of Fig. 6). In these examples, the differences between OCT–FFR and M-FFR were relatively small.

**Fig. 7:**
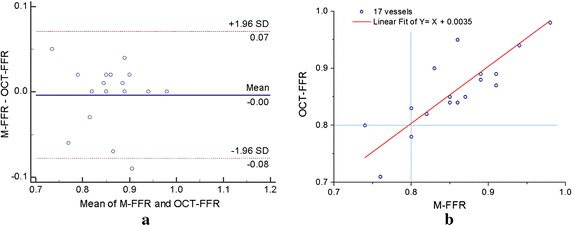
**a** Bland–Altman plot between clinically measured FFR (M-FFR) and OCT–FFR at the same location and **b** linear relationship between the M-FFR and the OCT–FFR with a coefficient of determination (R^2^) of 0.66, Pearson’s correlation coefficient (ρ_p_) of 0.82, and Spearman’s correlation coefficient (ρ_s_) of 0.80

Figure 7a shows a Bland–Altman plot between the clinically measured M-FFRs and the OCT–FFR values at identical locations; a mean difference of 0.00 and a 95% limit of agreement ranging from −0.07 to 0.08 (*n* = 17, standard deviation, SD = 0.04) was observed, indicating good agreement between M-FFR and OCT–FFR values. Figure 7b shows linear correlation with a coefficient of determination (R^2^) of 0.66; the OCT–FFR correlated strongly with clinically measured M-FFR, with a Pearson’s correlation coefficient (ρ_p_) of 0.82 and a Spearman’s correlation coefficient (ρ_s_) of 0.80 (*n* = 17; P < 0.001). Applying the cut-off level of M-FFR = 0.8 to the OCT-FFR resulted in three true positives, 13 true negatives, no false positives, and one false negative.

## Discussion

In this paper, we present an efficient and accurate method to perform OCT–FFR simulations by coupling a 3D CFD model from geometrically correct OCT images with a LPM based on vessel lengths extracted from CAG data. Simulated OCT–FFR values were compared to M-FFR values to validate the present method.

There were four main findings in this study. First, we proposed an efficient simulation method to solve coronary hemodynamics based on OCT images. OCT as an imaging modality can accurately generate lumen geometry of coronary arteries, and so has a strong potential for use in coronary hemodynamics simulation. Although there have been several computational methods of coronary arterial hemodynamics for patient-specific model reconstructed from CT images [2–4, 32–34], their methods were not extended to OCT–FFR simulation. Recently, Wang et al. [32] proposed a patient-specific model of coronary hemodynamics by coupling CFD with LPM. This method was applied to coronary hemodynamic simulations having bypass graft between coronary arteries [33, 34]. The computational method used in these studies is based on muscle mass method to estimate LPM parameter. On the other hand, we employed another novel method to estimate the resistance of LPM by measuring vessel length [25, 27, 28], because ventricular muscle mass cannot be determined from OCT images. The absence of LPM model causes a significant limitation for accurate hemodynamic computations. As discussed in Taylor et al. [4], assignment of unique resistance and capacitance values of the LPM for each outlet boundary is a key issue to compute more accurate coronary hemodynamic solutions. In this sense, the present method based on vessel length provided a useful approach to set the outlet boundary condition of the CFD model reconstructed from OCT images.

Second, the total resistance after the local vessel geometry of the OCT-derived ROI is introduced in Eq. (7). According to this equation, the resistance depends on the total resistance after the parent vessel and the resistance amplification factor. Here, the total resistance after the parent vessel can be obtained from the vessel length, as shown in Fig. 5c. In Eq. (7), the branched vessels before the ROI affect the resistance amplification factor, indicating that more vessel branching before the ROI increases the resistance amplification factor and eventually results in an increase in vessel resistance (Fig. 4). To our knowledge, there is no reported formulation or estimation method for the flow resistance after a local and small vascular region. Application of this method is not limited to the simulation of an OCT-derived model. Using this method for CT image-based FFR simulation, we can simulate only a small ROI model of coronary arteries extracted from CT images.

Third, although there were few clinically validated cases, comparisons between the simulated OCT–FFR values and clinically measured M-FFRs (Figs. 6, 7) showed that the present method can produce more accurate solutions than other studies. In the most recent clinical validation study, reported by Liu et al. [9], using a combination of OCT images and biplane CAG, the correlation coefficient and the SD of the Bland–Altman plot were 0.71 and 0.09, respectively. The study of OCT–FFR simulation by Tu et al. [6] resulted in a correlation coefficient of 0.8 and a SD of 0.06. Compared with these reports, the present study provided more accurate solutions, producing a correlation coefficient of 0.82 and a SD of 0.04 (Fig. 7). It is true that the degrees of stenosis in the simulated cases in this study were not severe. However, accurate simulated solutions would be expected even in cases with severe stenosis, because OCT images can provide very accurate lumen geometries, regardless of stenotic severity in the vessel ROI.

Fourth, the current OCT–FFR simulation is fast in terms of simulation time (Fig. 5). Segmentation of the entire coronary arterial tree is costly in most previous studies [2–5, 9, 23, 24], whereas segmentation of the small ROI from OCT images is all that is required in the present study. Thus, we were able to markedly reduce the image segmentation time that represents a large proportion of the total simulation time for the patient-specific hemodynamic model. Furthermore, the solving time for the 3D CFD model is also short because the small ROI reduces the number of mesh points required. A major benefit of this fast simulation is obtaining on-site solutions for coronary hemodynamics by clinicians.

The present study still has some limitations. First, the present method was validated only with 17 vessels. Here, only single stenosis cases were studied. A technique to establish LPM for serial multiple stenoses should be investigated for further study. Thus, more cases should be investigated to further validate the present OCT–FFR simulation. Second, a lack of correct curvature information was inevitable during OCT image processing and thus the curvature effect was not considered in the 3D CFD model. Third, the present method was only a fluid dynamics simulation assuming fixed wall boundaries of the CFD model, excluding the effects of vessel wall contraction or dilation. Fourth, the division ratio α in Eq. 1 was simply assumed in the present method. We considered RCA supplies blood to the muscles of LV as well as right ventricle (RV) and divided the RCA vessel length into two parts, the coronary vessels feeding RV and LV muscles. The division ratio α, the volume ratio of LV to RV, was assumed to be 3.46 as observed in clinical observation [19]. In a previous clinical study, the variation of α had a different range (P < 0.01) between males and females (3.64 ± 0.72 for males, 3.17 ± 0.43 for females, and 3.46 ± 0.66 for the composite group in 75 subjects) [19]. In all the cases, the variation of α was within ±30% of 3.46. In this study, we conducted a sensitivity analysis for 10 cases with the ±30% variation of α (2.42 ≤ α ≤ 4.5) by decreasing and increasing by 10, 20 and 30% of the standard α value, 3.46. The sensitivity analysis showed that FFR value changes due to the ±30% variation of α value were negligible (within ±0.006). However, this standard value of α could be incorrect in advanced heart disease, or heart failure. To determine a more accurate patient-specific value of α, clinical validations by using further sensitivity analysis should be performed. Besides, we only considered a general physiological observation that longer vessel feeds more muscle mass and thus has less flow resistance. But in rare cases, the resistance in long winding artery may be high. However, we believe that these limitations do not greatly affect the major findings of the study.

## Conclusions

OCT-FFR simulation was performed by coupling a geometrically correct OCT images-based 3D CFD and a CAG-based LPM model. The present study provided a novel formulation for the total resistance of LPM that is introduced to simulate a 3D CFD model of the ROI accurately. The calculated FFR values were compared with clinical measurement, showing a good agreement between them. Accurate simulated solutions could be obtained even in the cases with severe stenosis or calcified vessel, because OCT images can provide very precise lumen geometries, regardless of stenotic severity and vascular calcifications in the vessel ROI. Especially, the application of this method is not limited to the simulation of an OCT-derived model but can be extended to any clinical image-derived models e.g. commonly used CT image-based FFR simulation in a small ROI model. Moreover, the present method is fast regarding computational time, enabling clinicians to obtain solutions directly handled within the hospital.

## Abbreviations

FFR: fractional flow reserve
OCT: optical coherence tomography
CAG: coronary X-ray angiography
CT: computed tomography
MRI: magnetic resonance imaging
IVUS: intravascular ultrasound
M-FFR: clinically measured fractional flow reserve using a pressure wire
OCT–FFR: computed fractional flow reserve from OCT images
CT-FFR: computed fractional flow reserve
RCA: right coronary artery
LAD: left anterior descending artery
LCX: left circumflex artery
ROI: region of interest
3D: three dimensional
CFD: computational fluid dynamics
LPM: lumped parameter model
SBP: systolic blood pressure
DBP: diastolic blood pressure
HR: heart rate
SV: stroke volume
HCT: hematocrit

## References

[1] Nakamura D, Nishino S, Attizzani GF, Bezerra HG, Costa MA. Identification of vulnerable plaques. Cardiac Interv Today. 2015.

[2] Kim HJ, Vignon-Clementel IE, Coogan JS, Figueroa CA, Jansen KE, Taylor CA (2010). Patient-specific modeling of blood flow and pressure in human coronary arteries. Ann Biomed Eng.

[3] Min JK, Taylor CA, Achenbach S, Koo BK, Leipsic J, Nørgaard BL, Pijls NJ, De Bruyne B (2015). Noninvasive fractional flow reserve derived from coronary CT angiography: clinical data and scientific principles. JACC Cardiovasc Imaging.

[4] Taylor CA, Fonte TA, Min JK (2013). Computational fluid dynamics applied to cardiac computed tomography for noninvasive quantification of fractional flow reserve: scientific basis. J Am Coll Cardiol.

[5] Koo BK, Erglis A, Doh JH, Daniels DV, Jegere S, Kim HS, Dunning A, DeFrance T, Lansky A, Leipsic J (2011). Diagnosis of ischemia-causing coronary stenoses by noninvasive fractional flow reserve computed from coronary computed tomographic angiograms: results from the prospective multicenter DISCOVER-FLOW (diagnosis of ischemia-causing stenoses obtained via noninvasive fractional flow reserve) study. J Am Coll Cardiol.

[6] Tu S, Barbato E, Köszegi Z, Yang J, Sun Z, Holm NR, Tar B, Li Y, Rusinaru D, Wijns W, Reiber JH (2014). Fractional flow reserve calculation from 3-dimensional quantitative coronary angiography and TIMI frame count: a fast computer model to quantify the functional significance of moderately obstructed coronary arteries. JACC Cardiovasc Interv.

[7] Ellwein LM, Otake H, Gundert TJ, Koo BK, Shinke T, Honda Y, Shite J, LaDisa JF (2011). Optical coherence tomography for patient-specific 3D artery reconstruction and evaluation of wall shear stress in a left circumflex coronary artery cardiovascular. Eng Technol.

[8] Suo J, McDaniel M, Eshtehardi P, Dhawan SS, Taylor RW, Samady H, Giddens DP. 3D optical coherence tomography (OCT)—an investigation of intimal-medial thickness (IMT) and wall shear stress (WSS) in a patient’s coronary artery. Comput Cardiol. 2011:217–19.

[9] Liu L, Yang W, Nagahara Y, Li Y, Lamooki SR, Muramatsu T, Kitslaar P, Sarai M, Ozaki Y, Barlis P, Yan F, Reiber JH, Tu S (2016). The impact of image resolution on computation of fractional flow reserve: coronary computed tomography angiography versus 3-dimensional quantitative coronary angiography. Int J Cardiovasc Imaging.

[10] Li Y, Gutiérrez-Chico JL, Holm NR, Yang W, Hebsgaard L, Christiansen EH, Mæng M, Lassen JF, Yan F, Reiber JH, Tu S (2015). Impact of side branch modeling on computation of endothelial shear stress in coronary artery disease: coronary tree reconstruction by fusion of 3D angiography and OCT. J Am Coll Cardiol.

[11] Tu S, Holm NR, Koning G, Huang Z, Reiber JH (2011). Fusion of 3d qca and ivus/oct. Int J Cardiovasc Imaging.

[12] Tu S, Holm NR, Christiansen EH, Reiber JH (2012). First presentation of 3-dimensional reconstruction and centerline-guided assessment of coronary bifurcation by fusion of X-ray angiography and optical coherence tomography. JACC Cardiovasc Interv.

[13] Tu S, Xu L, Ligthart J, Xu B, Witberg K, Sun Z, Koning G, Reiber JH, Regar E (2012). In vivo comparison of arterial lumen dimensions assessed by co-registered three-dimensional (3D) quantitative coronary angiography, intravascular ultrasound and optical coherence tomography. Int J Cardiovasc Imaging.

[14] Hebsgaard L, Nielsen TM, Tu S, Krusell LR, Maeng M, Veien KT, Raungaard B, Terkelsen CJ, Kaltoft A, Reiber JH, Lassen JF, Christiansen EH, Holm NR (2015). Co-registration of optical coherence tomography and X-ray angiography in percutaneous coronary intervention. The does optical coherence tomography optimize revascularization (DOCTOR) fusion study. Int J Cardiol.

[15] Abbara S, Arbab-Zadeh A, Callister TQ, Desai MY, Mamuya W, Thomson L, Weigold WG (2009). SCCT guidelines for performance of coronary computed tomographic angiography: a report of the Society of Cardiovascular Computed Tomography Guidelines Committee. J Cardiovasc Comput Tomogr.

[16] Pijls NH, De Bruyne B, Peels K, Der Van, Voort PH, Bonnier HJ, Bartunek J, Koolen JJ, Koolen JJ (1996). Measurement of fractional flow reserve to assess the functional severity of coronary-artery stenosis. N Engl J Med.

[17] Tearney GJ, Regar E, Akasaka T, Adriaenssens T, Barlis P, Bezerra HG, Bouma B, Bruining N, Cho JM, Chowdhary S, Costa MA, de Silva R, Dijkstra J, Di Mario C, Dudek D, Falk E, Feldman MD, Fitzgerald P, Garcia-Garcia HM, Gonzalo N, Granada JF, Guagliumi G, Holm NR, Honda Y, Ikeno F, Kawasaki M, Kochman J, Koltowski L, Kubo T, Kume T, Kyono H, Lam CC, Lamouche G, Lee DP, Leon MB, Maehara A, Manfrini O, Mintz GS, Mizuno K, Morel MA, Nadkarni S, Okura H, Otake H, Pietrasik A, Prati F, Räber L, Radu MD, Rieber J, Riga M, Rollins A, Rosenberg M, Sirbu V, Serruys PW, Shimada K, Shinke T, Shite J, Siegel E, Sonoda S, Suter M, Takarada S, Tanaka A, Terashima M, Thim T, Uemura S, Ughi GJ, van Beusekom HM, van der Steen AF, van Es GA, van Soest G, Virmani R, Waxman S, Weissman NJ, Weisz G (2012). Consensus standards for acquisition measurement, and reporting of intravascular optical coherence tomography studies: a report from the International Working Group for Intravascular Optical Coherence Tomography Standardization and Validation. J Am Coll Cardiol.

[18] Lee KE, Kwon SS, Ji YC, Shin ES, Choi JH, Kim SJ, Shim EB (2016). Estimation of the flow resistances exerted in coronary arteries using a vessel length-based method. Pflugers Arch.

[19] Lorenz CH, Walker ES, Morgan VL, Klein SS, Graham TP (1998). Normal human right and left ventricular mass, systolic function, and gender differences by cine magnetic resonance imaging. J Cardiovasc Magn Reson.

[20] Schreiner W, Buxbaum PF (1993). Computer-optimization of vascular trees. IEEE Trans Biomed Eng.

[21] Kassab GS (2006). Scaling laws of vascular trees: of form and function. Am J Physiol-Heart Circ Physiol.

[22] Sherman TF (1981). On connecting large vessels to small. The meaning of Murray’s law. J Gen Physiol.

[23] Kwon SS, Chung EC, Park JS, Kim GT, Kim JW, Kim KH, Shin ES, Shim EB (2014). A novel patient-specific model to compute coronary fractional flow reserve. Prog Biophys Mol Biol.

[24] Lee KE, Kim GT, Lee JS, Chung JH, Shin ES, Shim EB (2016). A patient-specific virtual stenotic model of the coronary artery to analyze the relationship between fractional flow reserve and wall shear stress. Int J Cardiol.

[25] Sankaran S, Moghadam ME, Kahn AM, Tseng EE, Guccione JM, Marsden AL (2012). Patient-specific multiscale modeling of blood flow for coronary artery bypass graft surgery. Ann Biomed Eng.

[26] Seiler C. Collateral circulation of the heart. In Assessment of the human coronary collateral circulation. Berlin: Springer; 2009. p. 120–41.

[27] Schaper W, Schaper J (1993). Collateral circulation: heart, brain, kidney, limbs.

[28] Wilson RF, Wyche K, Christensen BV, Zimmer S, Laxson DD (1990). Effects of adenosine on human coronary arterial circulation. Circulation.

[29] Chatzizisis YS, Coskun AU, Jonas M, Edelman ER, Feldman CL, Stone PH (2007). Role of endothelial shear stress in the natural history of coronary atherosclerosis and vascular remodeling: molecular, cellular, and vascular behavior. J Am Coll Cardiol.

[30] Back LD, Radbill JR, Crawford DW (1977). Analysis of pulsatile, viscous blood flow through diseased coronary arteries of man. J Biomech.

[31] Dai N, Lv HJ, Xiang YF, Fan B, Li WM, Xu YW (2016). Three-dimensional modeling and numerical analysis of fractional flow reserve in human coronary arteries. Postepy Kardiol Interwencyjnej.

[32] Wang W, Mao B, Wang H, Geng X, Zhao X, Zhang H, Xie J, Zhao Z, Lian B, Liu Y (2016). Hemodynamic analysis of sequential graft from right coronary system to left coronary system. Biomed Eng Online.

[33] Zhao X, Liu Y, Li L, Wang W, Xie J, Zhao Z (2016). Hemodynamics of the string phenomenon in the internal thoracic artery grafted to the left anterior descending artery with moderate stenosis. J Biomech.

[34] Wang W, Liu Y, Zhao X, Xie J, Qiao A (2017). Hemodynamic-based long-term patency of different sequential grafting: a patient-specific multi-scale study. J Mech Med Biol.

